# Sex-specific BDNF and APOE ε4 genotype interactions on white matter hyperintensity volume

**DOI:** 10.1093/geroni/igaa057.3252

**Published:** 2020-12-16

**Authors:** Brandon Pitts

**Affiliations:** University of Kansas, Olathe, Kansas, United States

## Abstract

The apolipoprotein E (APOE) gene is the strongest genetic risk factor for late-onset Alzheimer’s disease (AD). One variant of the brain-derived neurotrophic factor (BDNF) gene also confers higher risk of AD. APOE and BDNF genotypes may have interactive effects on AD pathology. The aim of this study was to determine whether APOE and BDNF genotype differentially impact white matter hyperintensity volume (WMHV). We used data from 212 cognitively unimpaired individuals from the Alzheimer’s Disease Neuroimaging Initiative (ADNI) We used generalized linear models to predict WMHV from BDNF genotype and an interaction with APOE. Sex, age, education, vascular risk, and imaging-based amyloid load (Florbetapir SUVR) were used as covariates. . WMHV was derived by using the Lesion Segmentation Toolbox (LST) in SPM12 with a threshold of k = 0.15. We used the Hachinski Ischemic Scale to measure for vascular risk. We found no significant interaction of BDNF-APOE on WMHV (β = 0.40, 95% CI: (-0.39, 1.20), p = 0.32). In sex-stratified analyses the BNDF-APOE interaction was significantly associated with WMHV in males (β = 1.14, 95% CI:(0.17, 2.11), p = 0.02), but not in females (β = -0.37, 95% CI: (-1.47, 0.76), p = 0.50). For males, carriers of both BDNF Met and APOE ε4 alleles had the highest WMHV. Sex-specific differences in BDNF expression may be related to hormonal differences, and BDNF genotype might underlie these differences. Given the continued interest in using BDNF as a treatment target for AD, understanding complex genotype interactions may aid in future therapeutic studies.

